# Retraction Note: Protective role of silymarin in a mouse model of renal Ischemia–Reperfusion injury

**DOI:** 10.1186/s13000-018-0746-4

**Published:** 2018-09-12

**Authors:** Jian Tan, Jianpeng Hu, Yonghui He, Feilun Cui

**Affiliations:** Department of Urology, Affiliated People’s Hospital of Jiangsu University, Zhenjiang, 212013 China

## Retraction Note: Tan et al. BMC Diagnostic Pathology (2015) 10:198 DOI 10.1186/s13000-015-0436-4

This article [1] is retracted at request of the Editor. After publication of this article [1] concerns were raised regarding aspects of the methodology, including the choice of stains being inappropriate or inadequate to answer the research question. A discrepancy was also noted between the number of groups described in the methods and the number of groups presented in some of the figures. Furthermore the western blot images did not entirely correspond with the information reported in the results. For these reasons the conclusions of the study cannot be considered to be supported by the data. All authors agree to this retraction.
